# Discovery of the first macrolide antibiotic binding protein in *Mycobacterium tuberculosis*: a new antibiotic resistance drug target

**DOI:** 10.1007/s13238-017-0502-7

**Published:** 2018-01-19

**Authors:** Qingqing Zhang, Huijuan Liu, Xiang Liu, Dunquan Jiang, Bingjie Zhang, Hongliang Tian, Cheng Yang, Luke W. Guddat, Haitao Yang, Kaixia Mi, Zihe Rao

**Affiliations:** 1 College of Life Sciences, Nankai University, Tianjin, 300071 China; 2 College of Pharmacy, Nankai University, Tianjin, 300071 China; 3 CAS Key Laboratory of Pathogenic Microbiology and Immunology, Institute of Microbiology, Chinese Academy of Sciences, Beijing, 100101 China; 4 School of Life Sciences, Tianjin University, Tianjin, 300072 China; 5 School of Chemistry and Molecular Biosciences, The University of Queensland, Brisbane, QLD 4072 Australia; 6 Laboratory of Structural Biology, School of Medicine, Tsinghua University, Beijing, 100084 China; 7 Tianjin International Joint Academy of Biotechnology & Medicine, Tianjin, 300457 China

**Dear Editor**,

The prevalence of multidrug-resistant *Mycobacterium tuberculosis* (*M. tuberculosis*) is an increasing problem worldwide (Zumla et al., 2013; Dong et al., 2015). According to a 2014 World Health Organization (WHO) report, 480,000 individuals world-wide developed multidrug-resistant tuberculosis (MDR-TB) and more than 100 countries have cases of extensively drug-resistant tuberculosis (XDR-TB). Compared with drug-susceptible TB, MDR-TB and XDR-TB require prolonged therapeutic treatment with a combination of a number of second-line drugs (Chen et al., 2013). For patients where TB remains persistent despite prolonged therapy with second-line TB drugs, the add-on agents including bedaquiline and delamanid are recommended for salvage therapy (Günther, 2014; WHO, 2014; Shim and Jo, 2013).

The mechanisms of antibiotic resistance developed by bacteria are highly diverse (Alekshun and Levy, 2007), including protection of the antibiotic target by site-of-action mutations or by chemical modification of the target/target site (e.g., methylation in the ribosomal 23S rRNA by ErmMT methyl-transferase) (Buriankova et al., 2003). The direct modification or inactivation of antibiotics by specific enzymes (e.g., acetyltransferases, phosphotransferases) can be another resistance mechanism. Alternatively, reduced permeability or increased efflux (Black et al., 2014) of the drugs can also occur, thus preventing the drug from gaining access to the target. *M. tuberculosis* has adopted several of these strategies to become resistant to the most widely used antibiotics. One of these is the utilization of the ATP-binding cassette (ABC) transporters, which are drug efflux pumps that lower the concentration of antibiotics within the bacterium. Indeed, ABC transporters have been divided into three classes based on phylogenetic analysis (Dassa and Bouige, 2001): (i) the classical exporters (ii) importers which are composed of hydrophobic transmembrane domains and hydrophilic nucleotide-binding domains, and (iii) the type-II ABC proteins that lack transmembrane domains (Nunez-Samudio and Chesneau, 2013), some of which mediate antibiotic resistance (Kerr et al., 2005; Sharkey et al., 2016).

Bioinformatics analysis shows that *M*. *tuberculosis* possesses as many as 87 ABC containing proteins. Amongst these, Rv3197 is predicted to be a non-canonical ABC protein with an N-terminal extension (1–117), an ABC1 motif (118–232) and an aminoglycoside phosphotransferase (APH) motif (256–312) but lacking any transmembrane regions. The sequence characterization suggested Rv3197 might be an antibiotic transporter. In our preliminary study, we evaluated the effect of Rv3197 in antibiotic susceptibility by measuring the MICs of several antibiotics including isoniazid, rifampicin, erythromycin, streptomycin, ampicillin, chloromycetin and tetracycline by overexpression of Rv3197 in *Mycobacterium smegmatis* (*M. smegmatis*), which is commonly used in lab as a model bacteria for *M. tuberculosis*. Our results indicated that Rv3197 affected the susceptibility of erythromycin (Table S1).

Since a biosafety level 3 lab is needed to direct study of *M. tuberculosis* and the amino acid sequence of Rv3197 in *M. tuberculosis* is equivalent to Mb3320 in *M*. *bovis* BCG, a vaccine strain with low pathogenicity, we chose *M*. *bovis* BCG as a model for studying the function of Rv3197. Quantitative real-time PCR (qRT-PCR) analysis showed that the transcription level of *rv3197* increases by 3.6 fold when *M*. *bovis* BCG is treated with 6 mg/L erythromycin (Fig. 1A), showing that expression of *rv3197* is erythromycin-inducible. To verify the effect of Rv3197 on erythromycin susceptibility, we first made an Mb3320-deletion mutant strain, denoted Δ*rv3197*-BCG. This mutant strain grew at a similar rate to the wild-type strain in 7H9 medium (Fig. S1A), demonstrating that, under the conditions where no antibiotic is added, Rv3197 has no effect on cell growth. However, upon exposure to 2 mg/L of erythromycin, cell growth was reduced in Δ*rv3197*-BCG compared to wild-type BCG. This effect was partially reversed in a complemented pMV361-*rv3197*/Δ*rv3197*-BCG strain (Fig. 1B). In the presence of 3 mg/L of erythromycin, *M*. *smegmatis,* which contains the *rv3197* gene (pMV261-*rv3197*-mc^2^155) has a growth advantage over *M*. *smegmatis* that has an empty pMV261 vector (pMV261-mc^2^155) (Fig. 1C). Likewise, the *M*. *bovis* BCG pMV261-*rv3197* construct also showed a growth advantage over wild type in the presence of 4 mg/L of erythromycin (Fig. 1D). In contrast, no growth difference was detected between pMV261-*rv3197*-mc^2^155 and pMV261-mc^2^155 and between pMV261-*rv3197*-BCG and pMV261-BCG in the absence of erythromycin (Fig. S1B and S1C). These data demonstrate that Rv3197 is a factor responsible for erythromycin resistance in both *M*. *bovis* and *M*. *smegmatis*. Based on sequence similarity, this protein is also expected to perform a similar role in other *Mycobacteria* (e.g., *Mycobacterium tuberculosis*, *Mycobacterium kansasii*, and *Mycobacterium avium*). In consideration of its antibiotic resistance phenotype, we henceforth refer to this protein as macrolide antibiotic binding protein-1 (MABP-1), signifying it as the first characterization of a macrolide antibiotic binding protein in *Mycobacterium*.

**Figure 1:**
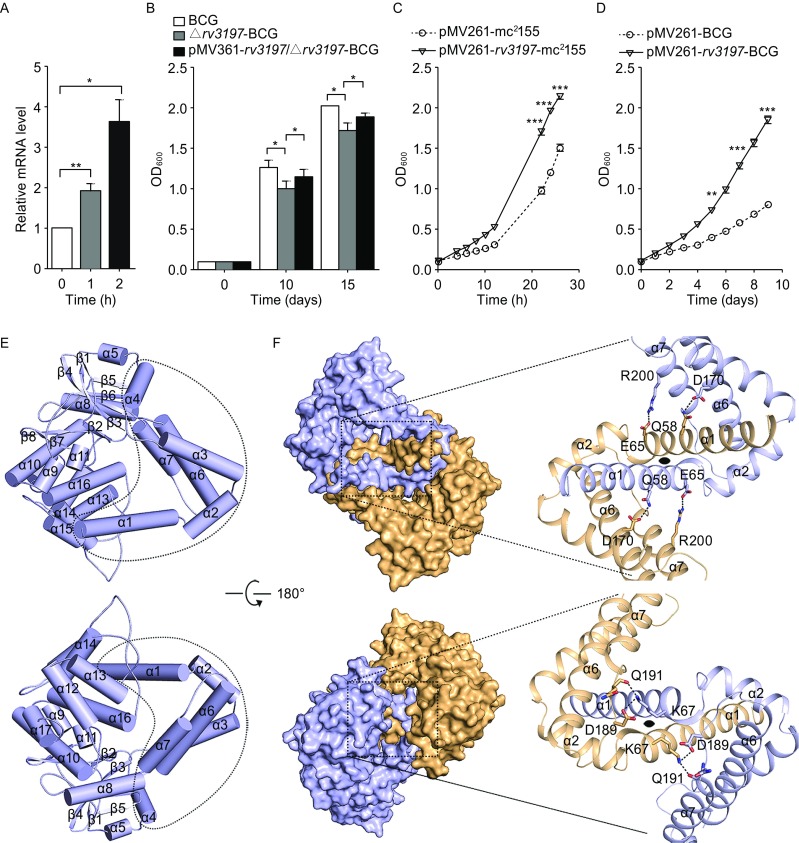
**The functional and structural studies of Rv3197 (MABP-1)**. MABP-1 confers inducible resistance to erythromycin in mycobacteria (A–D). (A) mRNA levels of *rv3197* in BCG determined by qRT-PCR after treatment with 6 mg/L erythromycin. (B) The growth of wild type BCG, Δ*rv3197*-BCG and pMV361*-rv3197*/Δ*rv3197*-BCG in 7H9 medium in the presence of 2 mg/L erythromycin. (C) Growth curves of pMV261-mc^2^155 and pMV261*-rv3197*-mc^2^155 in 7H9 medium in the presence of 3 mg/L erythromycin. (D) Growth curves of pMV261-BCG and pMV261*-rv3197*-BCG in 7H9 medium in the presence of 4 mg/L erythromycin. All results are shown as the mean ± standard deviation of three individual experiments. *P < 0.05, **P < 0.01, ***P < 0.001. The crystal structure of MABP-1 (E–F). (E) The domain arrangement of a MABP-1 monomer. The accessory domain is encircled by the dashed outline. (F) Left, surface representation of the MABP-1 dimer. Right, the dimer subunit interface. Each subunit is coloured differently. The α1 and α2 helices from each subunit insert into each other’s accessory domain. The black oval represents a two-fold axis perpendicular to the given view. Hydrogen bonds are shown as thick dashed lines

The crystal structure of apo-MABP-1 was determined at 2.2 Å by single wavelength anomalous dispersion (SAD) (Table S2). MABP-1 is a homodimer with each subunit containing a bilobal kinase domain (Fig. S2) and an accessory domain (Figs. 1E and S2). According to a DALI search the kinase domain strongly resembles that of an ancient UbIB protein kinase (ADCK3) (Table S3). The accessory domain consists of six α-helices: α1–α4 helices (residues 1–120) and α6–α7 helices (residues 161–206). These regions are insertions that are not found in the canonical kinase structures. Crucially, the α1 and α2 helices in each subunit insert into the accessory domain of its adjoining subunit, resulting in an N-terminal domain-swapped dimer (Fig. 1F). The accessible surface area that is buried upon dimerization is extensive, at 2021 Å^2^ per subunit.

Structural analysis confirmed that MABP-1 has an ATP binding loop (P-loop) in its kinase domain suggesting ATP as a substrate. Indeed, a spectrophotometric assay shows that it can hydrolyse ATP (*k*_cat_ = 0.0137 s^−1^, *K*_m_ = 0.285 mmol/L) (Fig. S3A), values that are comparable to that for other ABC transporters (Lin et al., 2015). Moreover, our data showed that the presence of erythromycin does not affect the ATPase activity of MABP-1 (Fig. S3B). To gain further insights into how MABP-1 recognizes ATP, the crystal structures of MABP-1 in complex with AMPPNP and with ADP, were determined. Since AMPPNP and ADP bind to MABP-1 in a similar fashion, only the binding mode of AMPPNP (Figs. 2A and S4) is discussed below. In this complex (Figs. 2B and S5A), AMPPNP is stabilized by extensive hydrogen bonds and hydrophobic interactions. The amino acids like S139, K157, E204, E210, Q242, E243, W244, I245 and D304 play important roles in ATP binding (Supplementary Materials). The ATPase activity of these mutants is investigated. Compared with the wild-type enzyme, the S139A and E210A mutants lost 75% and 82% activity, respectively (Fig. 2C), emphasizing their importance in nucleotide binding. The E204A and Q242A mutants retained 85% and 83% activity, respectively and the W244A mutant retained full activity compared to the wild-type (Table S4), suggesting lesser roles for these residues. Importantly, the S139A, K157A, E204A, E210A, Q242A and W244A mutants all have a significant decreased erythromycin resistant phenotype in *M*. *smegmatis* (Fig. 2D), suggesting that antibiotic resistance also depends on the ATPase activity of MABP-1.

**Figure 2:**
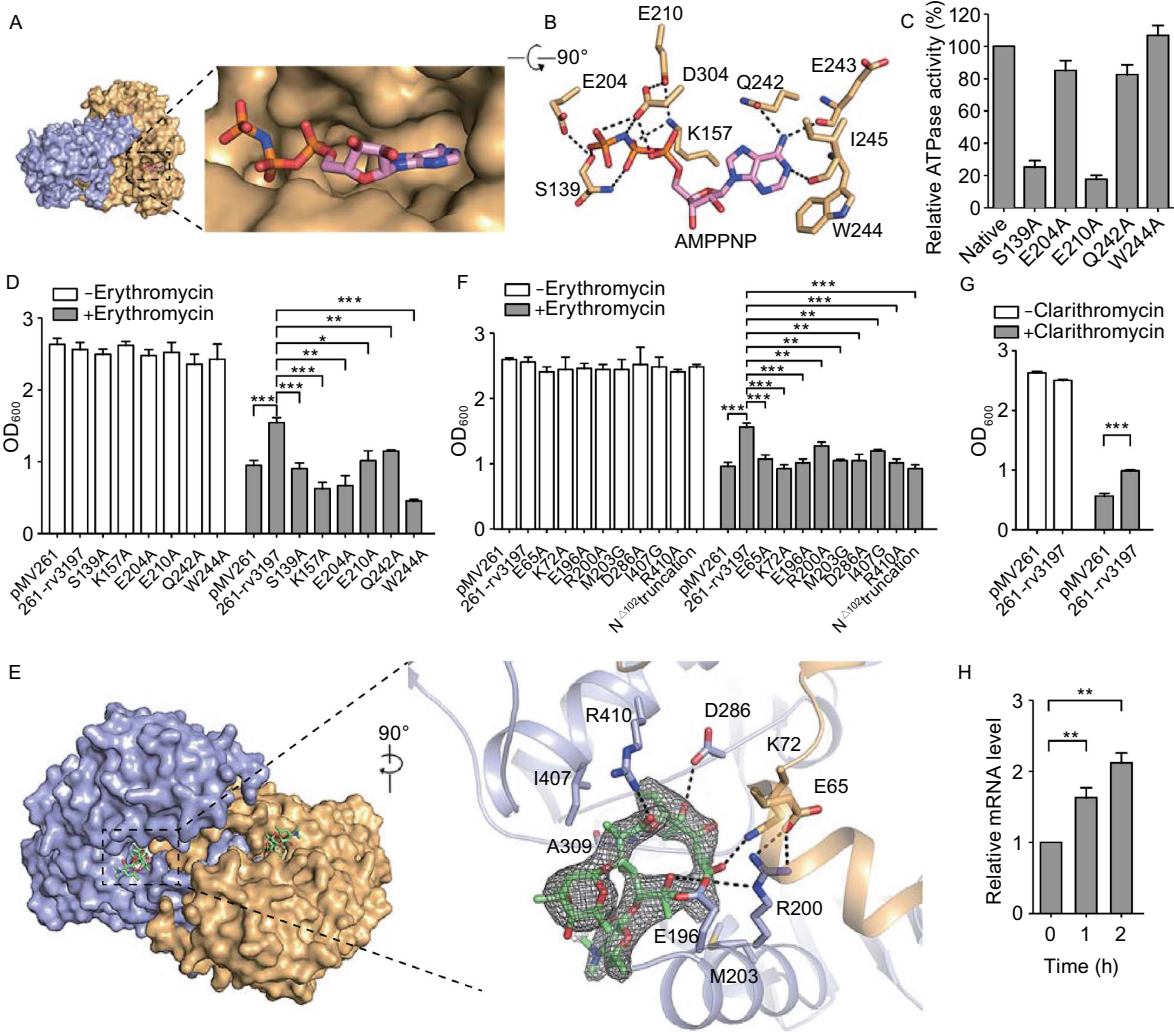
**MABP-1 is an ATPase and the accessory domain of MABP-1 is vital for erythromycin binding and dimerization**. (A) Structure of the MABP-1⋅AMPNP complex. MABP-1 is shown as a surface representation with AMPPNP as a stick model. Each subunit is coloured light orange and light blue. (B) The nucleotide-binding site in MABP-1. Hydrogen bonds are shown as dashed lines. (C) Relative ATPase activity of MABP-1 mutants in the nucleotide binding site (mean ± SD of three individual experiments). (D) The growth of mc^2^155 wild type and mutant strains in the presence (+) or absence (−) of 6.25 mg/L erythromycin (mean ± SD of three individual experiments, *P < 0.05, **P < 0.01, ***P < 0.001). (E) Left, surface representation of the erythromycin binding site. Erythromycin has green carbon atoms. Right, the *F*_*o*_ − *F*_*c*_ omit map for erythromycin, contoured at 3.0 *σ*. Residues that interact with erythromycin are shown as stick models. Hydrogen bonds are shown as dashed lines. (F) The growth of mc^2^155 wild type and mutant strains in the presence (+) or absence (−) of 6.25 mg/L erythromycin. (G) The growth of pMV261-mc^2^155 and pMV261-*rv3197*-mc^2^155 in 7H9 medium with (+) or without (−) 0.75 mg/L clarithromycin. (H) Quantification of mRNA levels of *rv3197* in BCG by qRT-PCR at the indicated time points after treatment with 1.5 mg/L clarithromycin (mean ± SD of three individual experiments, *P < 0.05, **P < 0.01, ***P < 0.001)

To understand how MABP-1 confers erythromycin resistance, the crystal structure of the complex was determined. The electron density for erythromycin was unequivocal revealing it is located in a shallow pocket formed by both subunits (Fig. 2E) where it is stabilized by extensive hydrogen bonds and hydrophobic interactions (Figs. 2E and S5B). Amongst these interactions are (i) a hydrogen bond between the carbonyl oxygen attached to C9 of the macrocyclic lactone ring (Fig. S6A) and the side-chain of K72 from the accessory domain of the opposing subunit, (ii) a hydrogen bond between the hydroxyl attached to C12 and the side-chain of D286, (iii) a hydrogen bond between the hydroxyl attached to C6 and the side chain of E196, (iv) a hydrogen bond between the carbonyl oxygen attached to C1 and the side chain of R410 and (v) hydrophobic interactions involving A309, I407 and M203 and the alkyl groups of the erythromycin (Fig. S5B). Two residues that contribute a significant portion of the surface of the erythromycin binding are E196 and R200, which are linked together by a salt bridge (Fig. 2E). As expected, the E65A, K72A, E196A, M203G, D286A, R410A mutants all significantly reduced the ability of MABP-1 to confer erythromycin resistance compared with wild-type *M*. *smegmatis* overexpressing MABP-1, emphasizing their critical roles in binding and in erythromycin resistance. In contrast, the R200A and I407G mutants produced only a slight reduction in the erythromycin resistance (Fig. 2F) while deletion of the entire N-terminal 102 residues (Rv3197^N△102^) completely abolished drug resistance in *M*. *smegmatis* (Fig. 2F).

To investigate macrolide specificity of MABP-1, clarithromycin (14-membered ring) and azithromycin (15-membered ring) were also assessed for their ability to inhibit the growth of *M*. *smegmatis* (Figs. 2G and S7). The data showed that overexpression of MABP-1 results in greater resistance to both erythromycin and clarithromycin than azithromycin, suggesting that MABP-1 has a preference for 14-membered ring macrolides rather than those with 15-membered rings (Supplementary Materials). The qRT–PCR data also shows that clarithromycin induces the upregulation of *rv3197* in BCG (Fig. 2H), again suggesting the 14-membered ring macrolides specificity of MABP-1.

In this study, we have identified a non-canonical ABC protein encoded by the *rv3197* gene in *M. tuberculosis*, as a macrolide antibiotic binding protein (MABP-1), which confers inducible resistance to the macrolide family of antibiotics. MABP-1 adopts an unusual three-dimensional fold with a kinase-like domain and a linked accessory domain. The accessory domain is indispensable for its function where it participates in dimerization of MABP-1 and in erythromycin binding. In addition, these data show that MABP-1 has a preference for 14-membered ring macrolides including erythromycin and clarithromycin rather than the 15-membered ring macrolides such as azithromycin.

Here we speculate that MABP-1 could dissociate erythromycin from the ribosome as has previously been suggested (Sharkey et al., 2016) in an ATP-dependent manner, reducing the concentration of erythromycin in the cytoplasm which is able to inhibit ribosomal activity. Another possibility is that MABP-1 is involved in modifying the structure of the antibiotic (Blair et al., 2015). The alternative hypothesis is that it cooperates with other efflux pumps to expel the drug (Nunez-Samudio and Chesneau, 2013). The structural comparison between MABP-1⋅AMPPNP and MABP-1⋅erythrmoycin may provide some clues to this hypothesis (Fig. S8 and Supplementary Materials). Clarification of the resistance mechanism of MABP-1 will require further investigation.

## Electronic supplementary material

Below is the link to the electronic supplementary material.

Supplementary material 1 (DOCX 2026 kb)
