# Supplementary material for: Discovery of the first macrolide antibiotic binding protein in Mycobacterium tuberculosis: a new antibiotic resistance drug target
Source: Protein Cell. 2018 Jan 19;9(11):971–5. doi: 10.1007/s13238-017-0502-7 (PMC6208485; doi:10.1007/s13238-017-0502-7)
Supplement: Supplementary file 1 — Supplementary material 1 (DOCX 2026 kb) [file 13238_2017_502_MOESM1_ESM.docx]

**Supplementary Discussion**

**The AMPPNP binding pocket of MABP-1**

The structural analysis of MABP-1·AMPPNP complex showed that AMPPNP is stabilized by extensive hydrogen bonds and hydrophobic interactions (Fig. 2B). The adenyl group is held in place by several key interactions. These include: (i) a π-stacking arrangement with the side chain of W244, (ii) a hydrogen bond between the N1 atom of the adenyl group and the carbonyl oxygen of I245, (iii) hydrogen bonds between the exocyclic N6 atom of the adenyl group and the side chain of Q242 and the carbonyl oxygen of E243. Holding the α and γ phosphate groups of the nucleotide in place are hydrogen bonds to D304, while the backbone amide of S139 from the P-loop forms a hydrogen bond with β-phosphate group, and the side chain of this residue forms a hydrogen bond with the γ phosphate group. The side chain of K157 forms a hydrogen bond with the β-phosphate group of AMPPNP (Fig. S5A).

**The macrolide specificity of MABP-1**

Erythromycin belongs to the macrolide class of antibiotics, which targets the 50S ribosome and inhibits bacterial protein synthesis ([Fair and Tor, 2014](#_ENREF_7)). As a second-generation macrolide, clarithromycin has been used against drug-resistant *M. tuberculosis* with synergistic activity in enhancing the activities of isoniazid, ethambutol, and rifampicin in MDR-TB and XDR-TB treatments ([Stephen J. Cavalieri et al., 1995](#_ENREF_20); [Bolhuis et al., 2013](#_ENREF_3); [Tae Sun Shim and Jo, 2013](#_ENREF_23); [Seung et al., 2014](#_ENREF_19)) and clinically against non-tuberculosis Mycobacteria ([van Ingen et al., 2012](#_ENREF_25); [Imran Ahmed et al., 2013](#_ENREF_8); [Jakko van Ingen et al., 2013](#_ENREF_10); [Philley and Griffith, 2013](#_ENREF_18); [Johnson and Odell, 2014](#_ENREF_11); [Tomonori Hirano et al., 2014](#_ENREF_24)).

The structural analysis of MABP-1·erythrmoycin shows that erythromycin is stabilized by extensive hydrogen bonds and hydrophobic interactions. Azithromycin differs from erythromycin (Fig. S6A) by a methyl-substituted nitrogen incorporated into its lactone ring (Fig. S6B), which could disrupt the hydrogen bond between the carbonyl oxygen of C9 and the side-chain of K72 of MABP-1. By contrast, in clarithromycin, the 6-O-methyl group (Fig. S6C) may still retain contact with MABP-1. Indeed, our data verify that MABP-1 has a preference for the 14-membered ring macrolides including erythromycin and clarithromycin rather than the 15-membered ring macrolides such as azithromycin (Fig. 2G-H). Thus, it is plausible that the 15-membered ring macrolides could be more potent antibiotics for the treatment of MDR-TB than 14-membered ring macrolides, though this assertion needs a comprehensive clinical investigation.

**The structural comparison between apo MABP-1 and MABP-1 in complex with AMPPNP and erythrmoycin**

The crystal structures of the MABP-1·AMPPNP and MABP-1·erythrmoycin complexes show there are no large-scale conformational changes to MABP-1 when either ligand binds, though there is a small change in conformation of the P loop (from residues 135 to 144) that holds AMPPNP in place when it binds, and a slight displacement of α7 helix（from residues 193 to 205）when erythromycin binds (Fig. S8). As a result a hydrogen bond (S139-E204) that connects these two regions of structure is broken when erythromycin is bound. Indeed, the two ligands are separated from each by only 8.9 Å when the complex structures are overlaid (Fig. S8), with the phosphate moiety pointing directly to the hydrophobic groups on erythromycin. Such an interaction has the potential to reduce the attraction between erythromycin and MABP-1, suggesting a scenario where this protein plays a role in removal of antibiotic from the cell is reasonable and that ATP may contribute to this process.

**Supplementary Materials and Methods**

**Bacterial strains and culture conditions**

Cultures of mycobacterial strains were grown in Middlebrook 7H9 medium (Becton Dickinson) supplemented with 0.5 % (v/v) glycerol, 0.05% (v/v) Tween 80 (Sigma) and 10% ADS enrichment (5% (w/v) bovine serum albumin fraction V, 2% (w/v) dextrose and 8.1%(w/v) NaCl), as previously described ([Li et al., 2015](#_ENREF_12)). Single bacterial colonies of *M. smegmatis* mc^2^155 strain were cultured on Middlebrook 7H10 medium (Becton Dickinson) containing 10% ADS, and 0.5% (v/v) glycerol. 7H11 media containing Middlebrook 7H11 medium (Becton Dickinson), 10% ADS, and 0.5% (v/v) glycerol was used for growing *M. bovis* BCG strains on agar plates. Kanamycin, erythromycin, azithromycin and clarithromycin were purchased from Sigma.

**Generation of knockout strain and overexpression strain of** ***rv3197* in *M. smegmatis* and *M. bovis* BCG**

Mycobacteriaphage-based specialized transduction was used to knockout *rv3197* in the *M. tuberculosis vaccine* strain *M. bovis* BCG ([Jain et al., 2014](#_ENREF_9); [Li et al., 2014](#_ENREF_13)). Briefly, the 5′ and 3′ flanking regions of *rv3197* were amplified from *M. bovis* BCG genomic DNA by the polymerase chain reaction (PCR) using the Rv3197LL/Rv3197LR and Rv3197RL/Rv3197RR primer pairs (primer sequences are listed in Table S5). The primers used had a PflMI site on the 5′ end to allow insertion into the pYUB1471 vector ([Stoyan Bardarov et al., 2002](#_ENREF_22)). The temperature sensitive phage phAE159 ([Stoyan Bardarov et al., 2002](#_ENREF_22)) and pYUB1471-*rv3197* were then digested with PacI and ligated using T4 DNA ligase to create a shuttle plasmid phAE159-*rv3197*. Phage packaging was performed using a MaxPlax packaging extract (Epicenter Biotechnologies, USA) according to the manufacturer’s instructions, and the shuttle plasmids were transformed into *E. coli* HB101 strain for amplification. The corrected plasmids were then transformed into *M. smegmatis* mc^2^155 by electroporation at 30°C for application of high titer phages. Gene knockout experiments were performed with a mixed high titer lysate and *M. bovis* BCG at an MOI of 10:1 at 37°C. The knockout clones were confirmed by PCR using the primer pairs Rv3197InL/Rv3197InR, Rv3197LLL/IL(R) and IR(F)/Rv3197RRR (Table S5). Complemented Rv3197 was constructed by cloning the full-length genes into the integrating vector, pMV361([Stover et al., 1991](#_ENREF_21)), to yield pMV361-*rv3197*/ Δ*rv3197*-BCG.

To overexpress the protein product of *rv3197* (MABP-1) in mycobacteria, the full-length coding gene of MABP-1 was amplified from *M. tuberculosis* H37Rv genomic DNA and cloned into the mycobacteria overexpression vector pMV261 ([Stover et al., 1991](#_ENREF_21)) to yield pMV261-*rv3197* for transformation into BCG (identified as pMV261-*rv3197*-BCG) and *M. smegmatis* mc^2^155, identified as pMV261-*rv3197*-mc^2^155. For a control, the empty pMV261 vector was also transformed into BCG (identified as pMV261-BCG) and into *M. smegmatis* mc^2^155, identified as pMV261-mc^2^155.

**Construction of MABP-1 point mutants and an N-terminal truncation**

Site-directed mutagenesis was performed using the Fast Mutagenesis System (Trans Gen Biotech) according to the manufacturer’s instructions. Primers for mutation of specific amino acids are shown in Table S5. The plasmids pMV261-*rv3197* and pGEX-6p-1-*rv3197* were used as the corresponding template to obtain serial mutants for overexpression in *M. smegmatis* and for protein purification in *E. coli*. The pMV261-*rv3197* mutants were transformed into *M. smegmatis* mc^2^155, and pGEX-6P-1-*rv3197* mutants were transformed into *E. coli* BL21-Codon Plus (DE3) RIL. The N-terminal truncation of 102 residues (Rv3197N^△102^) was amplified with the primers listed in Table S5, constructed into pMV261 and transformed into *M. smegmatis* mc^2^155 to obtain pMV261-*rv3197*N^Δ102^-mc^2^155.

**Testing for antibiotic resistance**

The inhibition curves under erythromycin treatment were determined in 50 ml 7H9 medium culture of recombinant *mycobacterial* strains in 100 ml flasks, by monitoring OD_600_ at indicated times, as described previously ([Li et al., 2015](#_ENREF_12)). Briefly, logarithmic phase *M. smegmatis* cultures (OD_600_ of 0.5) for all tested strains were diluted to an OD_600_ of 0.1 and then allowed to grow at 37°C with shaking at 200 opm in the presence of 3 mg/L erythromycin. The BCG strains were cultured at 37°C with shaking at 110 opm in the presence of 4 mg/L erythromycin. The growth of recombinant *M. smegmatis* in the presence of 0.75 mg/L clarithromycin was measured after 24 h incubation. All antibiotic resistance tests were repeated at least three times. Statistical analyses were performed using the standard t-test. *** *p* < 0.001, ** *p* < 0.01 and **p* < 0.05.

**Quantitative real-time PCR analysis**

Logarithmic phase cultures (OD_600_ of 1.8) of tested BCG strains were treated with 6 mg/L erythromycin and collected for RNA isolation, while untreated strains were set as control groups. The cell pellets were resuspended into TRIzol (Invitrogen, USA) and RNA was purified according to the manufacturer’s instructions. SuperScriptTM III First-Strand Synthesis System (Invitrogen, USA) was used for cDNA synthesis. Quantitative real-time PCR (qRT-PCR) was performed using 2x SYBR real-time PCR pre-mix (Takara Biotechnology Inc. Japan) in a Bio-Rad iCycler. The primers used for qRT-PCR are listed in Table S5. The mRNA levels of *rv3197* were normalized relative to the level of BCG *rpoD* (the coding sequencing of RNA polymerase sigma factor SigA). The relative gene expression was calculated using the 2^−ΔΔCT^ method ([Livak and Schmittgen, 2001](#_ENREF_14)). All assay experiments were repeated at least three times. Levels of mRNA expression of *rv3197* in BCG in the presence of 1.5 mg/L clarithromycin was carried out as described above.

**Cloning, expression and purification of MABP-1** **in *E. coli***

The gene encoding the full length MABP-1 was amplified by polymerase chain reaction (PCR) from *Mycobacterium tuberculosis* H37Rv genomic DNA and sub-cloned into prokaryotic expression vector pGEX-6p-1 with an N-terminal GST tag. Recombinant plasmid was transformed into *E. coli* BL21-Codon Plus (DE3) RIL for protein expression. Primers used in molecular cloning are shown in the Table S5.

Cells were grown in Luria Bertani medium supplemented with 100 mg/L ampicillin at 37°C until the OD_600_ reached 0.8 and then induced with 0.5 mM isopropyl-β-d-thiogalactopyranoside (IPTG) for 18 h at 16°C. Cells were harvested by centrifugation and lysed in 1 x PBS, pH 7.4 by ultrasonication. After centrifugation of cell lysates, supernatants containing soluble recombinant protein were loaded onto a GST-affinity chromatography (GE Healthcare) and then washed with 1 x PBS to remove non-specifically bound proteins. The GST tag was removed with PreScission Protease, and this MABP-1 was further purified with a Superdex 200 (10/300) gel filtration column (GE Healthcare) pre-equilibrated with a buffer containing 25 mM Tris-HCl, pH 8.0, and 150 mM NaCl. Fractions containing the target protein were then pooled and concentrated for crystallization. The expression and purification of the MABP-1 mutants used in the ATPase assay were carried out similarly as described above.

The selenomethionine (SeMet)-derivatized MABP-1 was expressed in the methionine-auxotrophic *E. coli* strain B834 (DE3) (Novagen) using M9 minimal medium supplemented with 60 mg selenomethionine/L. The purification of SeMet-derivatized MABP-1 was the same as that for the native protein.

**Crystallization and structure determination**

For crystallization, the purified MABP-1 was concentrated to 20 mg/mL in 25 mM Tris-HCl (pH 8.0), 150 mM NaCl. The protein solution for the apo MABP-1 was concentrated to 10 mg/ml. To prepare the ligand complex, MABP-1 (10 mg/ml) and each ligand (protein:ligand molar ratio of 1:5) were incubated overnight at 4°C before crystallization. Initial crystallization screening was performed using sitting-drop vapor-diffusion at 293K using crystal screening kits from Hampton Research and Wizard (Rigaku). Crystals of MABP-1 were observed after one week in a reservoir solution consisting of 2.0 M lithium sulfate, 0.1 M Tris (pH 8.5), and 2% (w/v) PEG 400. Crystals of the Se-Met derivative and the complexes with ADP and AMPPNP were obtained from the same conditions. Crystals in complex with erythromycin were obtained using a reservoir solution containing 1.8 M ammonium sulfate, 0.1 M Bis-Tris (pH 6.5), and 2% (v/v) polyethylene glycol monomethyl ether 550. All crystals were soaked in the reservoir solution supplemented with 25% (v/v) glycerol before being flash-cooled in liquid nitrogen.

Native diffraction data and SAD data were collected at 100 K on Beamline 5A of the Photon Factory (Japan). Diffraction data for the MABP-1·ADP and MABP-1·AMPPNP complexes were collected on Beamline 17U of the Shanghai Synchrotron Radiation Facility (SSRF). Data were processed with the HKL2000 suite of programs ([Otwinowski and Minor, 1997](#_ENREF_17)). Diffraction data for the MABP-1·erythromycin complex were collected on beamline 19U of the SSRF at 100K and were integrated, scaled and merged using the HKL3000 suite of programs ([Minor et al., 2006](#_ENREF_16)).

The structure of apo MABP-1 was determined by the single-wavelength anomalous diffraction (SAD) method from a Se-Met derivative protein at the peak wavelength. Se atoms were located and the initial phases were calculated using PHENIX AUTOSOL WIZARD([Adams et al., 2010](#_ENREF_1)). Automated model building was performed using the Autobuild module of PHENIX ([Adams et al., 2002](#_ENREF_2)) and then refined using the native data collected to 2.2 Å resolution. Manual adjustments of the model were made using COOT (version 0.7.1) ([Emsley et al., 2010](#_ENREF_6)) and refined using PHENIX(version 1.8.4).

The structures of MABP-1·ADP, MABP-1·AMPPNP and MABP-1·erythromycin were solved by molecular replacement with Phaser ([McCoy et al., 2007](#_ENREF_15)) in CCP4 ([Elizabeth Potterton et al., 2003](#_ENREF_5)) using the native structure as a search template. Refinement was performed in PHENIX with cycles of manual rebuilding in COOT. All structures were validated by MolProbity ([Chen et al., 2010](#_ENREF_4)). The statistics of the phasing and refinement are summarized in Table S2. The figures were prepared using PyMOL (DeLano Scientific) unless otherwise stated.

**ATPase assay**

ATPase activity was determined using QuantiChromTM ATPase/GTPase Assay Kit (BioAssay Systems). Reactions were performed in 96-well plates in a 40 μL volume with 20 μL of 2 x assay buffer (40 mM Tris, 80 mM NaCl, 8 mM MgAc2, 1 mM EDTA, pH 7.5), 10 μg of purified MABP-1, or one of the mutants. Reactions were initiated by the addition of 1 mM ATP. All measurements were performed at 25°C for 20 min in triplicate. To test the endogenous ATPase activity, a control with no enzyme was performed. 200 μL malachite green reagent was added to stop the reaction. This was followed by 30 min incubation at room temperature. The absorbance at 620 nm was measured using a microplate reader (BioTek, Synergy 4). The rate of ATP hydrolysis was then determined by assaying the liberated Pi, which is based on a standard curve. Data were then fitted to the Michaelis–Menten equation.

**References**

Adams, P.D., Pavel V. Afonine, Ga´bor Bunko´ czi, Vincent B. Chen, Ian W. Davis, Nathaniel Echols, Jeffrey J. Headd, Li-Wei Hung, Gary J. Kapral, Ralf W. Grosse-Kunstleve*, et al.* (2010). PHENIX: a comprehensive Python-based system for macromolecular structure solution. Acta Crystallogr D Biol Crystallogr 66, 213-221.

Adams, P.D., Ralf W.Grosse-Kunstleve, Li-Wei Hung, Thomas R. Ioerger, Airlie J. McCoy, Nigel W. Moriarty, Randy J. Read, James C. Sacchettini, and, N.K.S., and Terwilliger, T.C. (2002). PHENIX: building new software for automated crystallographic structure determination. Acta Crystallogr D Biol Crystallogr D58, 1948-1954.

Bolhuis, M.S., van Altena, R., van Hateren, K., de Lange, W.C., Greijdanus, B., Uges, D.R., Kosterink, J.G., van der Werf, T.S., and Alffenaar, J.W. (2013). Clinical validation of the analysis of linezolid and clarithromycin in oral fluid of patients with multidrug-resistant tuberculosis. Antimicrob Agents Chemother 57, 3676-3680.

Chen, V.B., Arendall, W.B., 3rd, Headd, J.J., Keedy, D.A., Immormino, R.M., Kapral, G.J., Murray, L.W., Richardson, J.S., and Richardson, D.C. (2010). MolProbity: all-atom structure validation for macromolecular crystallography. Acta Crystallogr D Biol Crystallogr 66, 12-21.

Elizabeth Potterton, Peter Briggs, Turkenburg, M., and Dodsona, E. (2003). A graphical user interface to the CCP4 program suite. Acta crystallographica Section D, Biological crystallography 59, 1131-1137.

Emsley, P., Lohkamp, B., Scott, W.G., and Cowtan, K. (2010). Features and development of Coot. Acta Crystallogr D Biol Crystallogr 66, 486-501.

Fair, R.J., and Tor, Y. (2014). Antibiotics and bacterial resistance in the 21st century. Perspect Medicin Chem 6, 25-64.

Imran Ahmed, Kauser Jabeen, and Hasan, R. (2013). Identification of non-tuberculous mycobacteria isolated from clinical specimens at a tertiary care hospital: a cross-sectional study. BMC Infectious Diseases 13.

Jain, P., Hsu, T., Arai, M., Biermann, K., Thaler, D.S., Nguyen, A., Gonzalez, P.A., Tufariello, J.M., Kriakov, J., Chen, B.*, et al.* (2014). Specialized transduction designed for precise high-throughput unmarked deletions in *Mycobacterium tuberculosis*. MBio 5, e01245-01214.

Jakko van Ingen, Beatriz E Ferro, Wouter Hoefsloot, Martin J Boeree, and Soolingen, D.v. (2013). Drug treatment of pulmonary nontuberculous mycobacterial disease in HIV negative patients the evidence. Expert Review of Anti-infective Therapy 11, 1065–1077.

Johnson, M.M., and Odell, J.A. (2014). Nontuberculous mycobacterial pulmonary infections. J Thorac Dis 6, 210-220.

Li, X., Li, J., Hu, X., Huang, L., Xiao, J., Chan, J., and Mi, K. (2015). Differential roles of the hemerythrin-like proteins of *Mycobacterium smegmatis* in hydrogen peroxide and erythromycin susceptibility. Sci Rep 5, 16130.

Li, X., Tao, J., Hu, X., Chan, J., Xiao, J., and Mi, K. (2014). A bacterial hemerythrin-like protein MsmHr inhibits the SigF-dependent hydrogen peroxide response in mycobacteria. Front Microbiol 5, 800.

Livak, K.J., and Schmittgen, T.D. (2001). Analysis of relative gene expression data using real-time quantitative PCR and the 2^-ΔΔCT^ Method. Methods 25, 402-408.

McCoy, A.J., Grosse-Kunstleve, R.W., Adams, P.D., Winn, M.D., Storoni, L.C., and Read, R.J. (2007). Phaser crystallographic software. Journal of Applied Crystallography 40, 658-674.

Minor, W., Cymborowski, M., Otwinowski, Z., and Chruszcz, M. (2006). HKL-3000: the integration of data reduction and structure solution--from diffraction images to an initial model in minutes. Acta Crystallogr D Biol Crystallogr 62, 859-866.

Otwinowski, Z., and Minor, W. (1997). Processing of X-ray Diffraction Data Collected in Oscillation Mode. Methods in Enzymology 276, 307-326.

Philley, J.V., and Griffith, D.E. (2013). Management of nontuberculous mycobacterial (NTM) lung disease. Semin Respir Crit Care Med 34, 135-142.

Seung, K.J., Becerra, M.C., Atwood, S.S., Alcantara, F., Bonilla, C.A., and Mitnick, C.D. (2014). Salvage therapy for multidrug-resistant tuberculosis. Clin Microbiol Infect 20, 441-446.

Stephen J. Cavalieri, Jon R. Biehle, and W. Eugene Sanders, J. (1995). Synergistic activities of clarithromycin and antituberculous drugs against Multidrug-Resistant *Mycobacterium tuberculosis*. Antimicrobial Agents and Chemotherapy 39, 1542–1545.

Stover, C.K., Cruz, V.F.d.l., Fuerst, T.R., Burlein, J.E., Benson, L.A., and Bennett, L.T. (1991). New use of BCG for recombinant vaccines. . Nature 351, 456-460.

Stoyan Bardarov, Svetoslav Bardarov Jr, Martin S. Pavelka Jr, Vasan Sambandamurthy, Michelle Larsen, JoAnn Tufariello, John Chan, Graham Hatfull, and Jr, W.R.J. (2002). Specialized transduction: an efficient method for generating marked and unmarked targeted gene disruptions in *Mycobacterium tuberculosis*, *M. bovis* BCG and *M. smegmatis*. Microbiology 148, 3007-3071.

Tae Sun Shim, and Jo, K.-W. (2013). Medical treatment of pulmonary multidrug-resistant tuberculosis. Infect Chemother 45, 367-374.

Tomonori Hirano, Minoru Matsuura, and Nakase, H. (2014). Pulmonary Mycobacterium avium Infection in a Patient with Crohn's Disease under Azathioprine Treatment. Case Rep Gastroenterol 8, 182-185.

van Ingen, J., Boeree, M.J., van Soolingen, D., and Mouton, J.W. (2012). Resistance mechanisms and drug susceptibility testing of nontuberculous mycobacteria. Drug Resist Updat 15, 149-161.

**
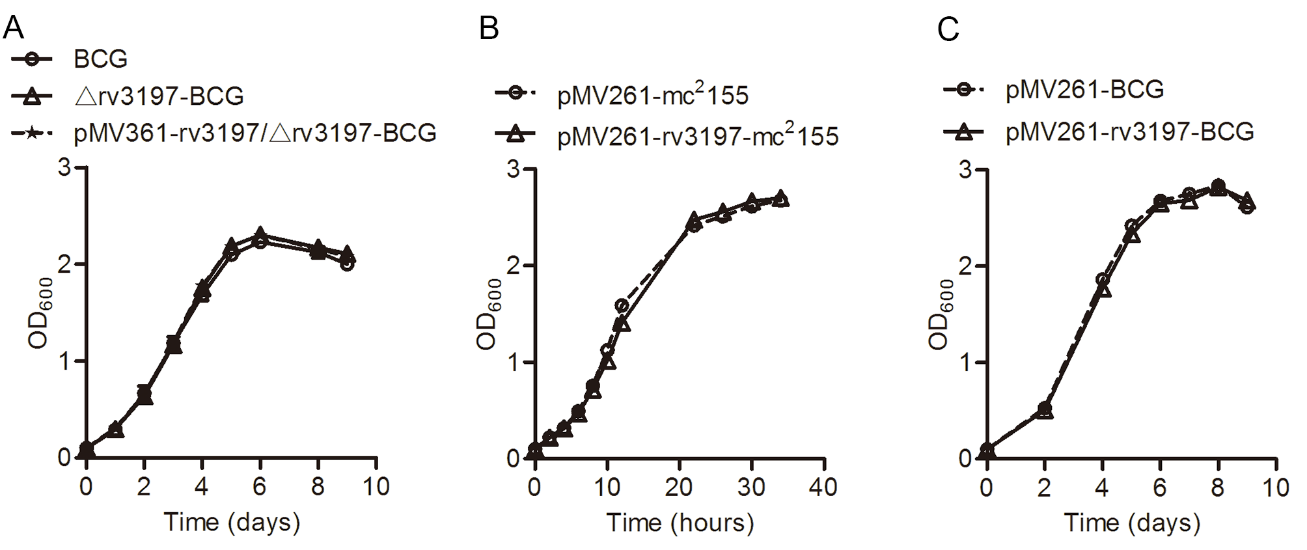
**

**Supplementary Figure 1.** Growth curves for BCG and mc2155 strains of Mycobacteria in 7H9 medium. (A) Wild type BCG, Δ*rv3197*-BCG and pMV361-*rv3197*/Δ*rv3197*-BCG. (B) pMV261-mc2155 and pMV261-*rv3197*-mc2155. (C) pMV261-BCG and pMV261-*rv3197*-BCG. All results are shown as the mean ± standard deviation of three independent experiments.


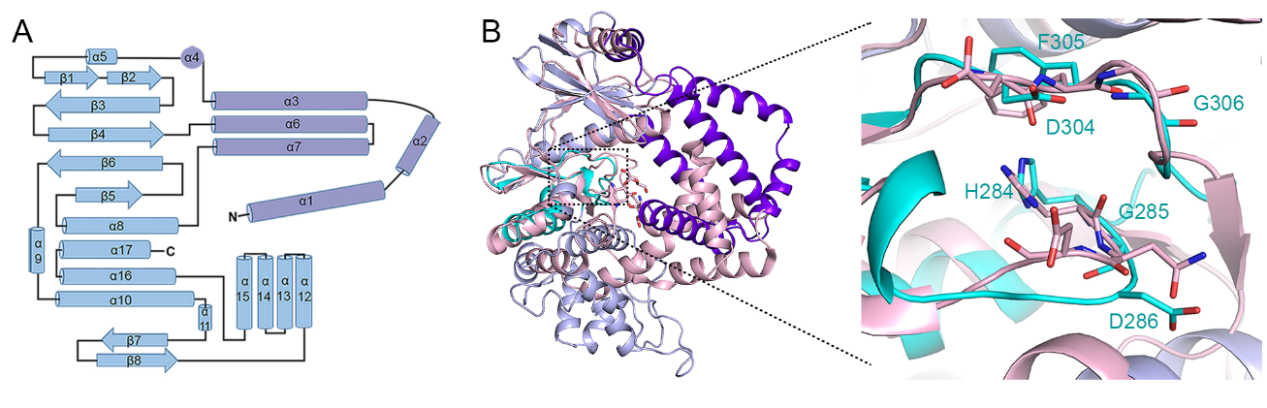


**Supplementary Figure 2.** The monemer of Rv3197 contains a bilobal kinase domain and an accessory domain. (A) Topology diagram of Rv3197 with the accessory domain highlighted in purple. (B) Structural comparisons between Rv3197(blue and purple) and aminoglycoside kinase APH(2’’)-IVa (pink, PDB:4dfu). The APH motif of Rv3197 is highlighted in cyan, the catalytic loop (HXD) and the activation segment(DXG) are shown as sticks.


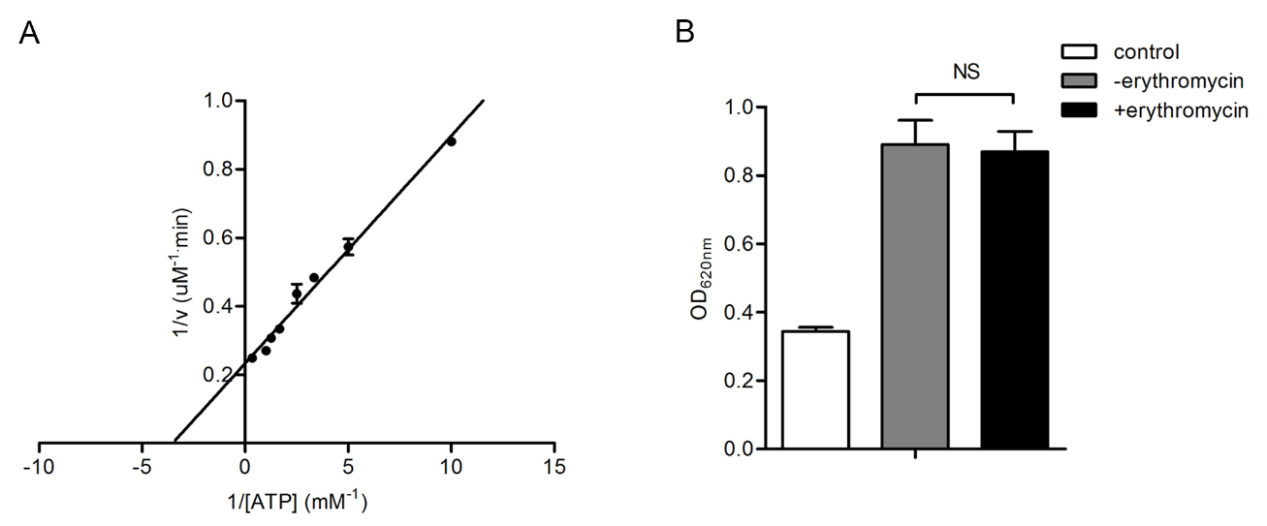


**Supplementary Figure 3.** The ATPase activity of MABP-1. (A) The Lineweaver-Burk plot of ATPase activity of MABP-1. MABP-1 hydrolyses ATP with a Michaelis constant (*K*m) of 0.285 mM and *k*cat of 0.0137 s-1 for ATP. All results are shown as the mean ± standard deviation of three independent experiments. (B) The ATPase activity of MABP-1 in the presence and absence of erythromycin. NS: no significance.


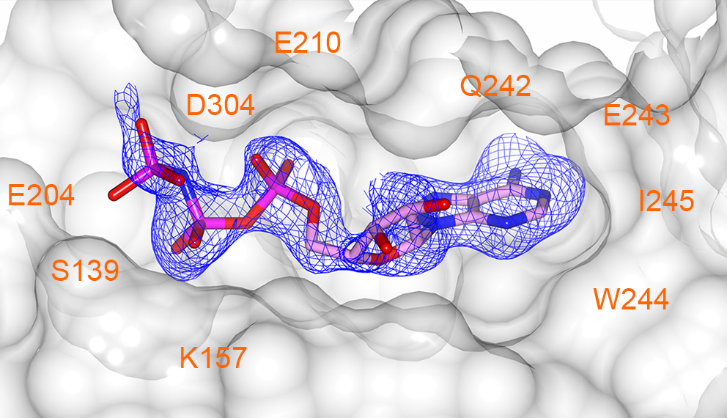


**Supplementary Figure 4.** The AMPPNP binding sites in MABP-1. The Connolly surface is in the background. The *Fo*-*Fc* omit map for AMPPNP are overlayed onto the ligand structure.


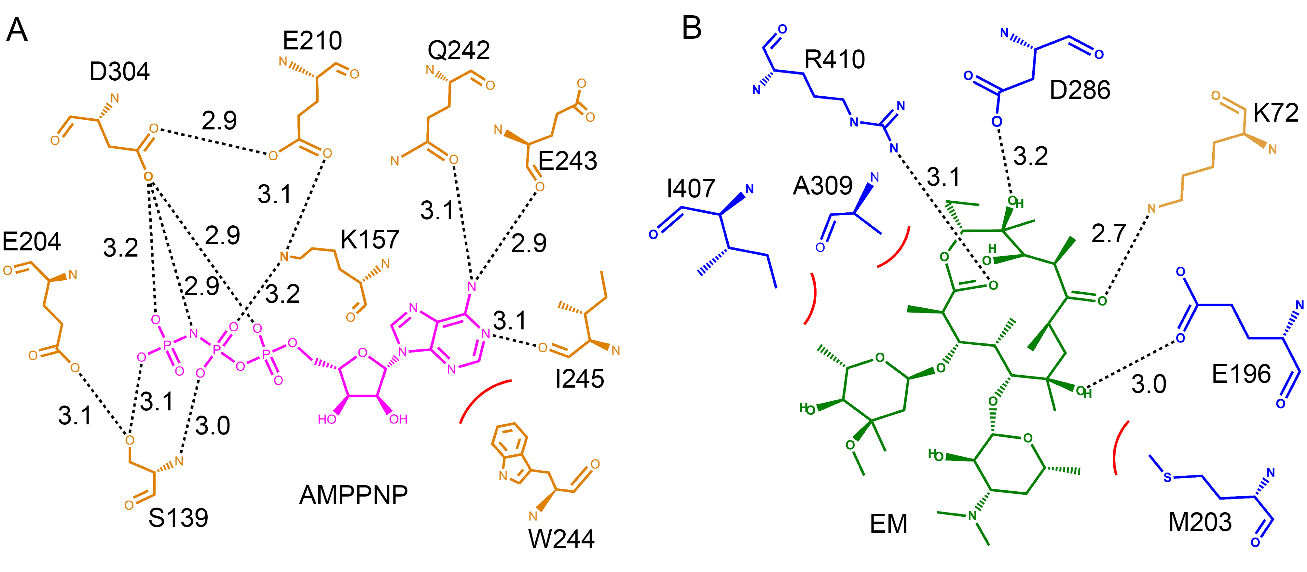


**Supplementary Figure 5.** Two dimensional representation of the interactions between MABP-1 and ligand. (A) The AMPPNP complex. (B) The erythromycin complex. Subunit A is in blue, subunit B is in orange. Hydrogen bonds are shown as black dashed lines with distances (Å) indicated. The red arcs represent hydrophobic interactions.


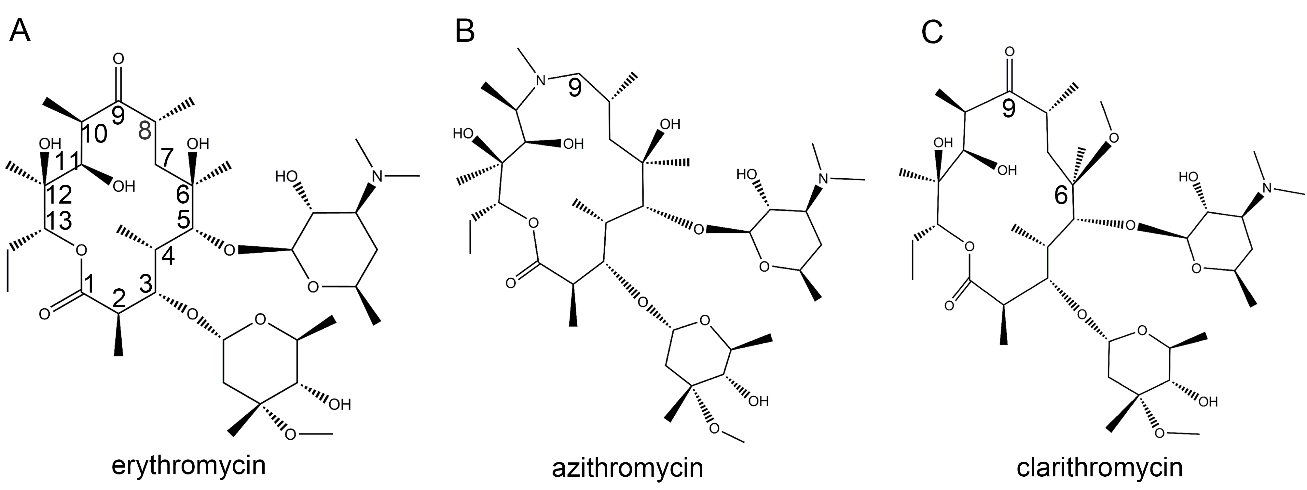


**Supplementary Figure 6.** The chemical structures of the three antibiotics in this study. (A) erythromycin, (B) azithromycin and (C) clarithromycin.


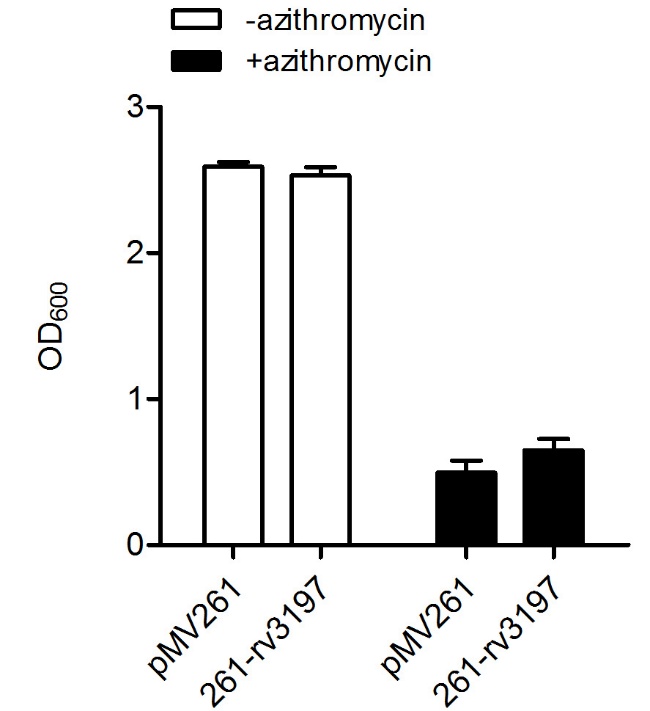


**Supplementary Figure 7.** Growth rates of pMV261-mc^2^155 and pMV261-*rv3197*-mc^2^155 in 7H9 medium with (+) or without (-) 3 mg/L azithromycin.

**
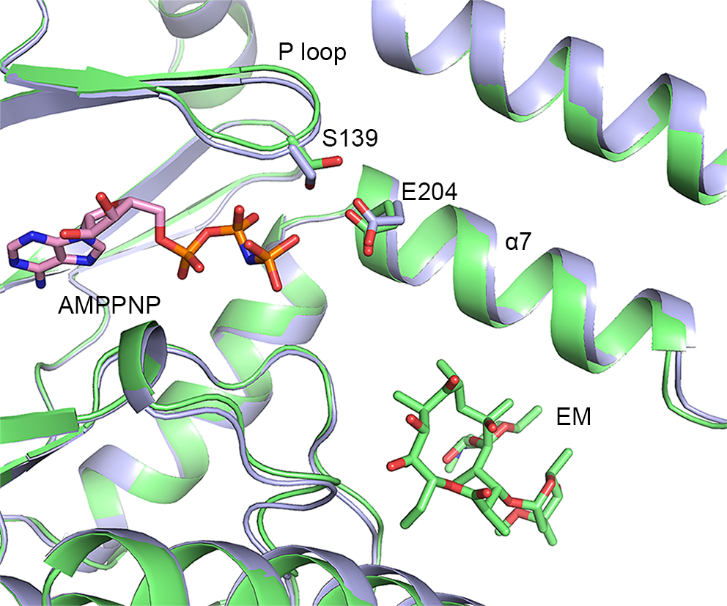
**

**Supplementary Figure 8.** Superimposition of the MABP-1·AMPPNP (blue) and MABP-1·erythromycin complexes (green). The two binding sites are separated by 8.9 Å. The image suggests that the binding of ATP can modulate the binding of erythromycin.

**Supplementary Table S1. MICs of erythromycin against *M. smegmatis* harboring the empty vector pMV261 and pMV261+Rv3197.**

| Strain | MIC (mg/L) |
| --- | --- |
| pMV261 | 25 |
| pMV261+ Rv3197 | 50 |

**Supplementary Table S2. Data collection and refinement statistics.**

|  | **Native MABP-1** | **MABP-1(Se-Met)** | **MABP-1**·**ADP** | **MABP-1**·**AMPPNP** | **MABP-1**·**EM** |
| --- | --- | --- | --- | --- | --- |
|  |  |  |  |  |  |
| Data collection |  |  |  |  |  |
| Space group | *P*6322 | *P*6322 | *P*6322 | *P*6322 | *P*6322 |
| Cell dimensions |  |  |  |  |  |
| a, b, c (Å) | 187.3 187.3 67.6 | 186.9 186.9 67.3 | 187.0 187.0 67.3 | 186.7 186.7 67.5 | 187.2 187.2 67.2 |
| ɑ, ß, γ (°) | 90 90 120 | 90 90 120 | 90 90 120 | 90 90 120 | 90 90 120 |
|  |  | Peak |  |  |  |
| Wavelength (Å) | 1.00849 | 0.97921 | 0.97906 | 0.97906 | 0.97855 |
| Resolution (Å) | 50-2.16  (2.20-2.16)a | 50-2.15  (2.19-2.15) | 50-2.10  (2.14-2.10) | 50-2.10  (2.14-2.10) | 50-2.80  (2.90-2.80) |
| *R*_merge_^b^ | 0.062(0.270) | 0.102 (0.460) | 0.090 (0.699) | 0.169 (0.786) | 0.079(0.723) |
| I /σ(I) | 45.8(8.2) | 49.9(3.6) | 27.9 (2.5) | 13.7(3.9) | 26.5(2.7) |
| Completeness (%) | 99.8(97.7) | 99.3 (87.5) | 99.9 (100.0) | 99.9 (100.0) | 99.4(100.0) |
| Redundancy | 14.3(12.1) | 13.5 (7.3) | 13.8(12.4) | 14.2(14.2) | 13.2(13.4) |
| Refinement |  |  |  |  |  |
| Resolution(Å) | 35.39-2.16 |  | 45.27-2.10 | 42.12-2.10 | 34.71-2.81 |
| No. reflections | 37811 |  | 40423 | 40493 | 17071 |
| *R*work/*R*free c | 0.177/0.208 |  | 0.191/0.228 | 0.190/0.216 | 0.229/0.274 |
| No. of atoms |  |  |  |  |  |
| Protein | 3120 |  | 3107 | 3122 | 3048 |
| Water | 338 |  | 342 | 208 |  |
| Ligand |  |  | 27 | 31 | 51 |
| B-factors |  |  |  |  |  |
| Protein | 33.0 |  | 33.5 | 38.0 | 62.2 |
| Water | 41.7 |  | 43.5 | 43.4 |  |
| Ligand |  |  | 41.9 | 59.2 | 70.4 |

| R.M.S. deviations |  |  |  |  |  |
| --- | --- | --- | --- | --- | --- |
| Bond lengths (Å) | 0.006 |  | 0.004 | 0.006 | 0.002 |
|  |  |  |  |  |  |
| Bond angles (°) | 1.035 |  | 0.868 | 0.967 | 0.721 |
|  | | | | | |
| Ramachandran  plot (%) | | | | | |
|  |  |  |  |  |  |
| Favored | 98.8 |  | 98.7 | 99.0 | 97.2 |
|  |  |  |  |  |  |
| Outliers | 0.25 |  | 0.00 | 0.00 | 0.00 |

^a^ Highest-resolution shell is shown in parentheses.

^b^ *R*_merge_ =*Σ_i_ |I_i_-‹I›| / Σ‹I›*, where *Ii* is an individual intensity measurement and *‹I›* is the average intensity for all the reflections.

^c^ *R*_work_=*Σ ||F_o_|-|F_c_|| / Σ|F_o_|*, where *F_o_* and *F_c_* are the observed and calculated structure factors, respectively.

**Supplementary Table S3. A search for structural homologues using the Dali server revealed that Rv3197 has kinase fold.**

| **No.** | **Protein Data Bank Chain** | **Z-score** | **RMSD** | **Identity**  **(%)** | **Name of protein** |
| --- | --- | --- | --- | --- | --- |
| 1 | 5i35-A | 26.9 | 4.1 | 25 | Atypical kinase ADCK3, Mitochondrial |
| 2 | 4ped-A | 24.0 | 4.8 | 25 | Atypical kinase ADCK3, Mitochondrial |
| 3 | 3re4-B | 13.1 | 3.6 | 17 | Rio-type serine/threonine-protein kinase Rio1 |
| 4 | 1zth-C | 13.1 | 3.2 | 15 | RIO1 serine protein kinase |
| 5 | 1zp9-C | 13.1 | 3.2 | 15 | RIO1 kinase |
| 6 | 5iqb-C | 12.9 | 3.4 | 14 | Bifunctional AAC/APH |
| 7 | 5iqh-D | 12.8 | 3.8 | 15 | Bifunctional AAC/APH |
| 8 | 1ztf-A | 12.7 | 3.9 | 16 | RIO1 serine protein kinase |
| 9 | 5iqe-C | 12.7 | 3.4 | 14 | Bifunctional AAC/APH |
| 10 | 4dfu-A | 12.6 | 3.5 | 15 | APH(2'')- IVa |

**Supplementary Table S4. ATPase activity data for wild type and mutant MABP-1.**

| Enzyme | Enzyme activity (μmol.min-1.mg-1) | Relative activity (%) |
| --- | --- | --- |
| Wild type | 243.05±5.34 | 100 |
| S139A | 60.93±7.48 | 25.07 |
| E204A | 206.87±9.83 | 85.11 |
| E210A | 42.91±5.19 | 17.66 |
| Q242A | 201.99±6.20 | 83.11 |
| W244A | 260.66±6.96 | 107.25 |

Errors represent the standard deviations from data measured in triplicate.

**Supplementary Table S5. Primers used in molecular cloning and mutagenesis. Restriction sites are underlined.**

| **Name** | **Sequence (5′–3′)** |
| --- | --- |
| **Rv3197-6p F** | CGGGATCCATGGATGATGGGAGTGTGTC |
| **Rv3197-6p R** | CGGAATTCTCAGACGACGATCGCGTC |
| **Rv3197-pMV361 F** | CCGGAATTCGATGATGGGAGTGTGTCAGA |
| **Rv3197-pMV361 R** | CCCAAGCTTTCAGACGACGATCGCGTCGG |
| **Rv3197-pMV261 F** | CGCGGATCCAGATGATGGGAGTGTGTCAGA |
| **Rv3197-pMV261 R** | CCCAAGCTTTCAGACGACGATCGCGTCGG |
| **Rv3197 truncation-pMV261**  **F** | CGGGATCCAGCCCCACCGCTGCCCGCCA |
| **Rv3197 truncation-pMV261**  **R** | CCCAAGCTTTCAGACGACGATCGCGTCGG |
| **E65A F** | GTTGTTTACCGTCCTCGGCGCACTCAAGGGTG |
| **E65A R** | GCGCCGAGGACGGTAAACAACTGATTGGCGGCC  T |
| **K72A F** | AAGGGTGGCGCGATGGCCGTCGGCCAGG |
| **K72A R** | GGCCATCGCGCCACCCTTGAGTTCGCC |
| **S139A F** | CCCAGTGGCATCTGCCGCCATCGGCCAGG |
| **S139A R** | GCGGCAGATGCCACTGGGGTGTCGTTGAACG |
| **K157A F** | ACGGCCGAGAAGTGGCCGTCGCGATCCAGTAT |
| **K157A R** | GCGACGGCCACTTCTCGGCCGTCCGACCAGAT |
| **E196A F** | CAAGGGGTGGTCGACGCACTGGTTGAAC |
| **E196A R** | GCGTCGACCACCCCTTGGACGTCGGC |
| **R200A F** | GACGAACTGGTTGAAGCCACCGAAATGG |
| **R200A R** | GCTTCAACCAGTTCGTCGACCACCCC |
| **M203G F** | GTTGAACGCACCGAAGGGGAACTCGACT |
| **M203G R** | CCTTCGGTGCGTTCAACCAGTTCGTC |

| **E204A F** | GAACGCACCGAAATGGCACTCGACTACC |
| --- | --- |
| **E204A R** | GCCATTTCGGTGCGTTCAACCAGTTCGTCGAC |
| **E210A F** | CTCGACTACCGGCTGGCGGCCGCCAACC |
| **E210A R** | GCCAGCCGGTAGTCGAGTTCCATTTCGGTGC |
| **Q242A F** | GCGCACCGAAGGTGGTGATCGCGGAGTGGATC |
| **Q242A R** | GCGATCACCACCTTCGGTGCGCTTGCCACGAC |
| **W244A F** | CGAAGGTGGTGATCCAGGAGGCGATCGAAGGTG |
| **W244A R** | GCCTCCTGGATCACCACCTTCGGTGCGCTTG |
| **D286A F** | GCTGGGGTTGATGCACGGCGCCGCCCACC |
| **D286A R** | GCGCCGTGCATCAACCCCAGCCGCCGTGGTG |
| **I407G F** | CCGGCCAAGCTCGCGGGTCCGATGCGGG |
| **I407G R** | CCCGCGAGCTTGGCCGGCAGGTCCAT |
| **R410A F** | CTCGCGATTCCGATGGCGGTTATCGCAT |
| **R410A R** | GCCATCGGAATCGCGAGCTTGGCCGG |
| **Rv3197 qF** | CCGCCAATCAGTTGTTTACC |
| **Rv3197 qR** | CTTCTGCAGCTTGGTCAGTG |
| **BCG RpoD qF** | TGATGACCGAGCTTAGCGAG |
| **BCG RpoD qR** | TTGGCTTCCAGCAGATGGTT |
| **Rv3197LL** | TTTTTTTTCCATAAATTGGTGGATCTGGTGGTGTT  GTCG |
| **Rv3197LR** | TTTTTTTTCCATTTCTTGGTATCCGGTCGGGGTGT  TGAT |
| **Rv3197RL** | TTTTTTTTCCATAGATTGGGATCAAGGCCCTGTC  GGAG |
| **Rv3197RR** | TTTTTTTTCCATCTTTTGGCAAACGCGCAGGTCAG  AAAA |
| **Rv3197InL** | GTCTTCCACTACACCCGCAA |
| **Rv3197InR** | CGATCTGTGGTTCGCCGATA |
| **Rv3197LLL** | AGGCATTGGAACGACTCGAA |

| **Rv3197RRR** | ATGAAGAGGCGTTGACCCAG |
| --- | --- |
| **IL(R)** | TCGACGACCCTAGAGTCC |
| **IL(F)** | GACACACCAACAGCATGGT |
